# Meeting of Am. Medical Association

**Published:** 1853-06

**Authors:** 


### EDITORIAL DEPARTMENT.

*Meeting of the American Medical Association.* — The sixth annual meeting of the American Medical Association commenced on Tuesday, the 3d ultimo, at the Bleecker street Presbyterian church, in the city of New York, and continued till the evening of the following Thursday. As was anticipated, the attendance was large, larger than at any previous meeting. Nearly six hundred delegates and members were assembled. The states of New

York, Massachusetts, and Pennsylvania, were the most numerously represented, and, relatively, in the order just enumerated. But very few of the states of the Union were without representatives.

Having had the pleasure of being present during the session, we should feel bound to give the readers of this Journal as full an account of the proceedings, etc., as practicable, were it not that several of the New York daily newspapers have contained very complete reports, which, it is to be presumed have been perused by the great majority of the members of the profession throughout the country. This being the case, we shall content ourselves, with a concise summary of the more important of the measures adopted. In noticing these, we will follow the chronological order of their adoption.

### *First Day.*

The city of St. Louis, Mo., was with great unanimity designated as the place for the next annual meeting of the Association.

In accordance with the recommendation of the publishing committee, the annual subscription was raised to $5. This was deemed necessary in order to cover the expense of the publication of the annual volume of Transactions.

The President of the Association, Dr. Wellford, of Virginia, read an address, which was received with marked approbation, a vote of thanks being taken, and a copy requested for publication in the annual volume of Transactions. While we shall find much to admire in this address, there will be room for critical remark when it appears in print. We refer more particu larly to the disparagement of the American medical profession, and of medical education in this country, in which the venerable writer conforms to a custom that we desire to see honored in its breach rather than observance. In compensation for this, as it seems to us, exceptionable part of the address, the benefits which have resulted from the Association were set forth with cogency and eloquence.

A communication from Dr. Ramsay, of Ga., was laid upon the table without reading. It was understood that the communication related to a complaint of a personal nature of the action of the Association at a previous meeting. Without knowing aught of the merits of the controversy in which Dr. Ramsay has been engaged, this action of the Association seemed to us to savor of injustice. We think the communication should have been read before being laid upon the table; but as the course pursued met with nearly an unanimous assent, we are bound to conclude that in the opinion we have expressed we are probably in the wrong.

The nominating committee (consisting of one delegate from each of the states represented at the meeting) reported the following names for officers for the ensuing year. The report was adopted, and the officers duly elected by resolution:

*President* — Dr. Jonathan Knight, of Conn.

*Vice-presidents* — Drs. Usher Parsons, of Long Island; Lewis Condit, of New Jersey; Henry R. Frost, of South Carolina, and H. L. Howard, of Ohio.

*Secretaries* — Drs. E. L. Beadle, of New York, and Edwin L. Lamoine, of Mo.

*Treasurer* — Dr. D. F. Condie, of Penn.

*Second Day.— Morning Session.*

The stated reports from the standing committees appointed at the last annual meeting, were called for in order. Several committees reported progress, and desired to be continued. Others did not respond to the call. Some sent in their declination. A small proportion only of the reports provided for, were submitted. Some of these were referred to the committee on publication, the titles alone being read; of others a synopsis was read, and a few, not being voluminous, were read in full.

The following is a list of the reports received and referred to the committee on publication:

1. On Acute and Chronic Diseases of the Neck of the Uterus. By Dr. Charles D. Meigs, of Penn.
   Referred without reading.

2. On the Agency of the Refrigeration produced by the Upward Radiation of Heat as an Exciting Cause of Disease. By Dr. G. Emerson, of Penn.
   A synopsis read by Dr. E.

3. On Typhoid Fever. By Dr. R. H. Campbell, of Ga.
   A verbal abstract presented.

4. On the Epidemics of Tennessee and Kentucky. By Dr. W. L. Sutton, of Kentucky.
   A synopsis submitted.

5. On Medical Education. By Dr. Pitcher, of Michigan.
   This was read in full.

At this stage of the proceedings the report of the committee on prize dissertations was called for. The committee reported an award of one of the two prizes to Dr. Waldo J. Burnett, of Boston, Mass., for a treatise on "The Cell, its Physiology, Pathology, and Philosophy." The second of the prizes was awarded to Dr. Washington L. Atlee, of Philadelphia, for a treatise on "The Surgical Treatment of certain Fibrous Tumors of the Uterus heretofore considered beyond the resources of art."

A voluntary communication on Diseases of the Hip Joint, by Prof. Alden March, of Albany, was favorably reported on by the committee, and the author was requested to read his paper, after the meeting, at the Crosby Street Medical School.

*Afternoon Session.*

1. Dr. Gordon Buck, of New York, read a paper on Morbid Growths within the Larynx, which was referred to the committee of publication.

The committees, and individuals to whom special subjects were assigned for reports to be presented at the next annual meeting were appointed, but we are not now prepared to give their names and a list of the subjects. This we will do in a future number.

*Third Day.—Morning Session.*

1. A Report on Medical Literature. By Dr. N. S. Davis, of Illinois. This interesting and able report was read in full.

2. "On the Results of Surgical Operations for the Relief of Malignant Diseases." By Dr. S. D. Gross. An abstract was read by Dr. L. P. Yandell.

Resolutions commemorative of the decease of the late Dr. Drake, of Cincinnati, of Dr. Isaac Parrish, and Dr. Horner, of Philadelphia, were adopted.

*Afternoon Session.*

A committee of five, (Dr. J. O. Edwards, chairman,) was appointed to report on the best mode of preventing the domestic adulteration of drugs.

The following resolutions submitted by Dr. Stille, of Philadelphia, were adopted:

1. That it is recommended to the several medical colleges, and such other boards as are by law authorized to examine candidates for admission to the medical profession, to require from every candidate or licentiate his signature to the code of ethics.

2. It is further recommended that the formal administration of a pledge faithfully to observe and keep the said code form part of the public exercises of medical commencements.

The subject of the amendments to the constitution came up at this stage of the proceedings. After considerable discussion the only amendments adopted were, first, to receive delegates from the army and navy in the proportion of one to ten of the number of medical officers in each branch of the science; and, second, to receive representatives from the American Medical Association in Paris.

Prior to adjournment, the Association adopted resolutions of thanks to the committee of arrangements, and to various institutions and citizens of New York, from whom courtesies had been received. A vote of thanks was also given to the press of the city for the accurate reports of the proceedings.

The recent meeting of the Association, we believe, is admitted to have been certainly not inferior in interest to any of the preceding meetings. The session throughout was characterized by harmony and good feeling. Of the scientific results a better opinion can be formed after the appearance of the volume of Transactions. We reserve remarks on the character of the papers which were read, either entire, or by abstracts, till they are to be perused in print. In our estimation, the meetings would possess greater interest if more time were accorded to scientific subjects, and less to medical schools, quackery, etc. An interchange of views on topics pertaining to the several departments of medical knowledge, (if we may judge others by ourselves,) would be far more attractive and edifying than the discussions which, at the last meeting, as heretofore, have consumed most of the meeting. Moreover, it seems to us this improvement would conduce to the dignified character of the Association. The *cacoethes loquendi* exhibited by a few members is a misfortune which is not peculiar to medical assemblages. This is a topic of general complaint, and is an evil not easily remedied; but we reckon it would be less annoying in proportion as the proceedings of the Association were to assume more exclusively a scientific character.

It remains to refer to the hospitalities extended to the members by the committee of arrangements, the profession of the city of New York, and other of the citizens of the great metropolis. We are at a loss how to dispose of this part of our duty. It was much easier to appreciate the social enjoyments and festivities of the memorable week of the session, than it is now to render them justice with our pen. The members were entertained each night as follows:

*Tuesday evening*—By Hon. Hamilton Fish, Dr. Isaac Wood, Dr. Willard Parker, and Dr. James R. Wood.

*Wednesday evening*—Hon. A. C. Kingsland, Dr. Delafield, Dr. Anderson, Dr. Detmold, and Dr. Isaac E. Taylor.

*Friday evening*—By Dr. Cheeseman, Dr. John Watson, Dr. Horace Green, and Dr. Lewis A. Sayre.

On Friday, a steamboat excursion took place, enabling the members to visit the public institutions at Staten Island, Randall's Island, and Blackwell's Island; and at the latter they were met and entertained by the Governors of the Almshouse.

To the various public institutions in the city, together with several private exhibitions of art and curiosity, the ticket of membership of the Association was equivalent to a card of admission.

But the crowning event was the banquet given by the physicians of the city on Thursday evening, at Metropolitan Hall. It is probably not exaggeration to say that no public entertainment ever given in the United States excelled this in profusion and splendor. A description of this festive occasion we leave to those competent to do rhetorical justice to such a theme. Or, let the reader imagine a thousand guests seated in the magnificent Metropolitan Hall, decorated with flowers; the galleries filled with ladies; brilliant music; the tables loaded with rich viands, and the anticipated delicacies of the summer; that which "maketh glad the heart of man" flowing in abundance; wisdom and wit from the lips of some of the most gifted members of our profession—here are the materials for the groundwork on which fancy may weave a picture, which, be it ever so highly colored, will hardly exceed the reality.

But amidst the vicissitudes of human existence, sorrows and joys often alternate in quick succession. On the morning after the joyful reunion to which we have just referred, occurred the terrible accident at Norwalk, by which several worthy members of the association, who were hastening to resume their active duties at home, were hurried into eternity. Among those lost by this memorable disaster were Dr. Peirson, of Salem, Mass.; Dr. Welch, of Hartford, Conn.; Dr. Beach of Bridgeport, Conn.; Drs. James M. Smith and John Gray, of Springfield, Mass.; Dr. Bartlett, of New Hampshire, and Dr. Wm. C. Dwight, of Moscow, N. Y.

Such events, albeit they may be attributable to human instrumentality, seem to be designed by Providence to remind us of the uncertain tenure of human life—a solemn truth, which, notwithstanding the number of such Divine admonitions, is so apt to be drowned by the pleasures and absorbing interests of this world.

